# Family-Based Obesity Prevention Interventions among Hispanic Children and Families: A Scoping Review

**DOI:** 10.3390/nu13082690

**Published:** 2021-08-03

**Authors:** Erica G. Soltero, Armando Peña, Veronica Gonzalez, Edith Hernandez, Guisela Mackey, Chishinga Callender, Jayna M. Dave, Debbe Thompson

**Affiliations:** 1 USDA/ARS Children’s Nutrition Research Center, Department of Pediatrics, Baylor College of Medicine, 1100 Bates St., Houston, TX 77030, USA; soltero@bcm.edu (E.G.S.); edith.hernandez@bcm.edu (E.H.); guisela.mackey@bcm.edu (G.M.); chishinga.callender@bcm.edu (C.C.); jmdave@bcm.edu (J.M.D.); 2 Center for Health Promotion and Disease Prevention, Arizona State University, 500 N. 3rd St., Phoenix, AZ 85004, USA; apena3@asu.edu; 3 Health Promotion and Health Education, School of Public Health, University of Texas Health, 1200 Pressler St., Houston, TX 77030, USA; VeronicaGonzalezRDN@gmail.com

**Keywords:** obesity, children, adolescents, social determinants of health, prevention interventions

## Abstract

This scoping review examined intervention and sample characteristics of family-based obesity prevention interventions among Hispanic youth. This review also examined the degree to which existing interventions were culturally-adapted, acknowledged social determinants of health (SDoH), and collaborated with community stakeholders. A comprehensive search across Medline Ovid, Embase, Scopus, PsycInfo, and Pubmed was used to identify 13 studies primarily based in the U.S. (92.3%). Data was extracted by two independent reviewers. Most used a randomized control trial design (69.2%), a behavior change theory (84.6%), and reported moderate to high (≥70%) retention (69.2%). Studies targeted improvements in physical activity (69.2%) and fruit and vegetable intake (92.3%) through nutrition education, cooking demonstrations, and tastings. Younger children from low socioeconomic backgrounds (61.5%) were well represented. Most interventions were culturally-adapted (69.2%), all studies reported collaboration with stakeholders, yet only half used strategies that acknowledged SDoH (46.2%). To increase our understanding of the underlying mechanisms by which family-based approaches can reach and engage Hispanic youth and families, future studies should rigorously evaluate theoretical constructs, family processes, and SDoH that influence program participation and health behaviors. This information will guide the design and development of future interventions aimed at reducing obesity disparities among Hispanic youth.

## 1. Introduction

Hispanic youth are disproportionately burdened by obesity (25.8% vs. 18.5% general population) and have experienced significant increases in severe obesity (12.4% vs. 7.9% general population) in the past decade [1]. The high prevalence of obesity in this population contributes to disparities in obesity-related disease risk factors including hypertension, dyslipidemia, and impaired glucose tolerance [2,3,4]. Hispanic youth with obesity also experience significant psychosocial consequences including reduced quality of life, social isolation, and body image dissatisfaction due to weight-based stigma and discrimination, compared to youth without obesity [5,6,7]. By 2050, Hispanics will represent 29% of the U.S. population, with Hispanic youth representing the fastest and largest growing pediatric subgroup [8]. It is a public health imperative to address obesity disparities in this growing, high-risk youth population, which has the potential to promote health equity and reduce disparities in obesity-related diseases [9].

Family-based interventions are recommended as a strategy for reaching and engaging Hispanic youth and families in obesity prevention [10]. The role of families and parents in the health and development of children has been well established [11,12]. Family-based approaches acknowledge that dietary habits and physical activity in youth are shaped and influenced within the context of familial processes and relationships [13,14,15]. Given that familismo or familism is a strong cultural value among Hispanics that places a strong emphasis on family and family commitment [10], obesity prevention interventions that are family-focused may be more effective in this population [15,16]. However, there are few guidelines and theoretical frameworks to guide the implementation and evaluation of family-based interventions, limiting our understanding of what makes these interventions effective or their long-term impact [9,17,18].

Recent reviews of obesity prevention interventions found that the most effective interventions for addressing obesity disparities among minority youth are those that are culturally-adapted, acknowledge social determinants of health (SDoH), and include multi-level community collaborations [9,15,19,20]. Culturally adapted interventions have been shown to increase engagement among Hispanics [15]. Cultural adaptations include peripheral strategies such as conducting language translations of materials and using culturally-appropriate images [9]. It also extends to ‘deep structure’ strategies that include the integration of broader social and cultural factors such as cultural values, norms, and traditions [9,21]. In addition to culture, obesity-prevention interventions among Hispanics should also acknowledge other SDoH that impact obesity-related outcomes and behaviors [22]. SDoH are the conditions in which people live, worship, work and go to school [23]. Hispanic youth and families are disproportionately burdened by inequitable experiences across obesity-related SDoH including limited access to healthcare, public services, and employment opportunities [22,23,24]. Developing intervention strategies that acknowledge SDoH requires collaborative research approaches given the breadth and complexity of SDoH [25,26]. Collaborating with community stakeholders can leverage community insights on priority determinants and existing resources for addressing SDoH [27,28,29].

Despite growing obesity disparities, obesity-prevention programs among Hispanic children remains a significant gap in the literature [15,16,19]. Therefore, the purpose of this scoping review is to systematically examine intervention and sample characteristics of studies that have implemented family-based obesity prevention interventions among Hispanic youth and families. The secondary purpose of this review is to examine the degree to which interventions are culturally-adapted, acknowledge SDoH, and collaborate with stakeholders for implementation. Specific objectives of our review are to describe intervention and sample characteristics to describe the current state of the science on family-based obesity prevention interventions among Hispanic youth and families. Based on an extensive review of the literature, we will summarize the findings, identify knowledge gaps, and highlight important areas of inquiry for future family-based obesity prevention strategies among Hispanic youth and families.

## 2. Methods

A scoping review is an appropriate methodology for reviewing large bodies of literature to generate an overview of a research topic [30]. This study was conducted using the five-stage methodological framework for scoping studies developed by Arksey and O’Malley [31]. Guided by this framework, the stages of this scoping review included: (1) identifying the research question; (2) identifying relevant studies; (3) selecting studies; (4) charting the data; and (5) synthesizing and summarizing the results. Methodology used for each of the stages within the framework are outlined below.

### 2.1. Identifying the Research Questions

The following research questions guided this review: (1) What are the intervention and sample characteristics of family-based pediatric obesity prevention programs among Hispanic/Latino children? (2) Do existing family-based strategies consider SDoH to meet the needs of Hispanic families? (3) To what extent do existing family-based strategies with Hispanic families involve collaboration among community stakeholders?

### 2.2. Identifying Relevant Studies

Relevant studies were defined as empirical, peer-reviewed articles that described a family-based obesity prevention intervention among Hispanic youth and families. A literature search of Medline Ovid was conducted using MeSH headings (obesity, pediatric obesity, overweight, pediatrics, adolescent, child, Hispanic Americans, parents, mothers, fathers, family), a floating MeSH subheading (prevention), and equivalent keywords and phrases. The search strategy is provided in Supplementary File Table S1. This same strategy was then applied to additional databases including Embase, Scopus, PsycInfo, and Pubmed.

### 2.3. Selecting Studies

Relevant studies published between 2010–2020 were identified during the search, screened by blinding the results to only show the title, and abstract and then screened using the Endnote (Clarivate^TM^, Philadelphia, PA, USA) referencing software. For the articles that met eligibility criteria, the full article was retrieved and assessed by two independent reviewers to ensure a consistent application of the eligibility criteria for inclusion in the review. Disagreements about study eligibility of the sampled articles were discussed between the two reviewers until a consensus was reached. Articles were selected if they described an intervention that included at least one parent or guardian over the age of 18 years and healthy children between the ages of 5–18 years with at least 75% of the population self-identifying as Hispanic/Latino, which could include Mexican, Mexican American, Spanish, Cuban, Puerto Rican, or South American. The study must also include a focus on obesity-related health behaviors including one or more of the following: improving diet (increasing fruit and vegetable intake, reduced fat and sugar intake, etc.), increasing physical activity, reducing sedentary behaviors or screen time, or improving sleeping habits. There were no requirements for sample size, child weight status, study location, or intervention setting. Articles were excluded if they: (1) did not involve parents or if the parent component was optional; (2) were not written in English; (3) were protocol studies or non-intervention studies (e.g., cross-sectional studies, qualitative studies, review articles); or (4) had overlap with another study.

### 2.4. Chart the Data

A ‘narrative review’ approach was applied to extract information from all studies [32]. Our extraction framework included 21 categories that aligned with the Preferred Reporting Items for Systematic reviews and Meta-Analyses extension for Scoping Reviews (PRISMA-ScR) Checklist and our research questions. Extraction categories and availability of data is presented in Supplementary File Table S2. Two independent reviewers extracted information from each article across each data extraction category. Reviewers met to compare extracted information; discrepancies were discussed between the two reviewers until a consensus was reached.

### 2.5. Synthesize and Summarize the Results

Descriptive statistics (i.e., frequencies) were calculated for intervention characteristics, sample characteristics, SDoH, and collaboration with stakeholders. A content analysis approach was used to summarize patterns found in the information extracted across data extraction categories [31]. Data synthesis and summation was focused on answering the research questions.

## 3. Results

**Figure 1:**
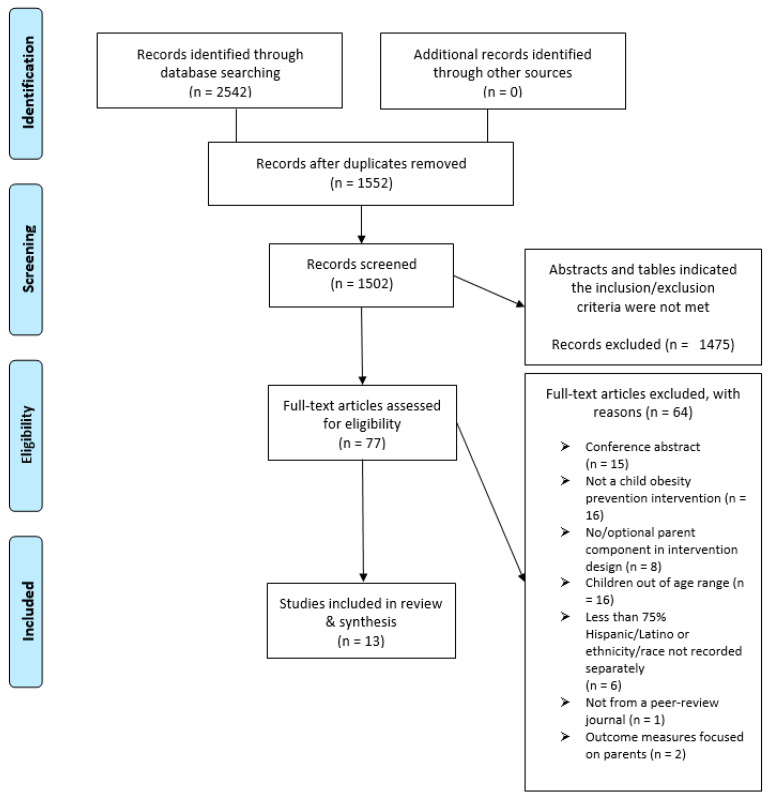
Consort diagram.

The search yielded a total of 2452 results, 1552 after de-duplication. The consort diagram in Figure 1 summarizes the review process. A total of 1475 papers were eliminated by the blind screening of titles and abstracts. Sixty-four papers were eliminated after reading the literature for various reasons outlined in the consort diagram. Some of the main reasons for elimination included: not a child obesity prevention intervention, optional or no parent component in intervention design, children out of age range, less than 75% Hispanic participants or ethnicity/race not reported. The search yielded a total of 13 papers published between 2010–2020 were included in this scoping review.

### 3.1. Intervention Characteristics

Table 1 provides a summary of intervention characteristics. Most studies were based in the U.S. (n = 12, 92.3%), and only one study was based in Mexico (7.7%).

While the Social Cognitive Theory (SCT) was the most widely used theory (n = 6, 46.2%), multiple ecologic and sociocultural theories were reported with several studies reporting the use of more than one behavior change theory. A more in-depth review of articles revealed that only three studies measured theoretical constructs, two of which used the SCT and Family Systems Theory (FST), and only one of these studies examined theoretical constructs as mediators. The most common study design was a randomized controlled trial (n = 9, 69%), followed by quasi-experimental designs (n = 5, 38.5%). There was great variability in intervention setting with primary care/health clinics (n = 3, 23.1%), community organizations (n = 2, 15.4%), the home (n = 2, 15.4%), and school (n = 2, 15.4%) setting having fairly equal representation. A few interventions were described as pilot studies lasting less than or equal to 10 weeks or less (n = 3, 23.1%), with just over half of studies lasting from 11–52 weeks (n = 7, 53.8%), and few studies lasting longer than one year (n = 3, 23.1%). Studies lasting longer than a year typically included a follow-up period. Of note, all interventions included a delivery format that included in-person, group-based didactic sessions. Almost all studies focused on improving dietary habits (n = 12, 92.3%) including improving fruit and vegetable intake and decreasing fat or sugar intake. Diet components were primarily delivered through the use of nutrition education (n = 9, 69.2%), food demonstrations (n = 2, 15.4%), food exposures (n = 3, 23.1%), or a focus on improving nutrition knowledge or literacy (n = 7, 53.8%; i.e., reading nutrition labels, portion size knowledge). Most studies also targeted increases in physical activity (n = 9, 69.2%), with fewer studies targeting sedentary behaviors (n = 6, 46.2%), reductions in screen use (n = 2, 15.4%), or sleep (n = 1, 7.7%). All studies evaluated study outcomes among children with 11 (85%) studies also including a parent-focused assessment. Finally, almost half of studies reported retention rates above 70% (n = 9, 69.2%), while fewer studies (n = 4, 30.8%) reporting poor retention rates below 70%. 

Given that this is a scoping review, an in-depth analysis of results and outcomes was not conducted [30]. However, our narrative review found that only four (31%) studies reported statistically or clinically significant improvements in BMI or BMIz scores. Additionally, four (31%) studies reported improvements in diet-related behaviors or knowledge, with just three (23%) studies reporting significant improvements in physical activity.

### 3.2. Sample Characteristics

Participant characteristics are presented in Table 2. All interventions included children between 5–10 years old (n = 13, 100%) or 11–13 years old (n = 9, 69.2%). Sample sizes varied, with smaller studies including 0–100 participants (n = 4, 30.8%) and larger studies including over 300 participants (n = 4, 30.8%). While several studies did not present data on family socioeconomic status (SES; n = 5, 38.5%), almost half reported that they focused on families with low SES (n = 5, 38.5%). Only three studies (23.1%) included a measure of acculturation. Most interventions were focused on engaging the entire family (n = 10, 76.9%), which included both parents and often included siblings; however, three studies (23.1%) focused on parent-child dyads only. While parent-child dyad studies provided stronger definitions for the parent/guardian and child or children to be included in the intervention, most interventions did not provide a clear definition of ‘family.’ For example, some studies reported that all siblings and family members within the home were encouraged to attend intervention sessions while others did not provide any clarification on the family members included in intervention sessions. All studies focused on families self-identifying as Hispanic/Latino, which primarily included families of Mexican or Mexican American decent (n = 10, 76.9%) as well as families from Central America (n = 3, 23.1%), Latin America (n = 2, 15.4%), or Latino/Hispanic non-specific (n = 5, 38.5%).

### 3.3. Integration of SDoH and Community Collaboration

The data extracted on SDoH as well as stakeholder collaboration are presented in Table 3. The most common SDoH included culture, language, and familial contextual factors. Most studies (n = 9, 69.2%) reported that intervention material was culturally adapted which included integrating cultural values and beliefs into curriculum content, use of cultural media (e.g., telenovelas), and adapted resources like food recipes and workbooks. Similarly, most studies reported that intervention content and materials were delivered in Spanish and English (n = 8, 44.4%), with only three (23.1%) being delivered solely in Spanish. About half of studies (n = 6, 46.2%) also addressed contextual factors that typically affect health behaviors and program participation among Hispanic families including stress from immigration, access to healthcare, and barriers to engaging in health behaviors promoted in the intervention. In regard to community collaboration, about half of studies (n = 7, 53.8%) included some sort of formative work in the form of qualitative interviews or the use of a community advisory board. Last, most studies collaborated with stakeholders for implementation including physicians/providers (n = 4, 30.8%) or health educators/promotoras (n = 6, 46.2%).

## 4. Discussion

Family-based obesity prevention interventions are recommended for addressing growing obesity disparities in Hispanic youth, highlighting a need to develop a better understanding of effective family-based strategies for engaging this population [9]. This review used rigorous systematic methods to conduct a scoping review of family-based obesity prevention interventions among Hispanic youth and families. In addition to examining intervention and sample characteristics, we examined the degree to which interventions considered SDoH and collaborated with stakeholders for intervention development and implementation. Studies included in this review included numerous strengths including the use of theoretically-driven, rigorous RCT study designs with moderate to high retention rates. Families from low SES backgrounds were well represented, providing support for the feasibility of family-based approaches to reach and engage high-risk families. Furthermore, most studies acknowledged the importance of addressing culture as most studies used culturally adapted materials delivered in Spanish or Spanish and English. There was also a high level of community collaboration as the majority of studies collaborated with stakeholders for implementation across clinic, community, and school settings. Despite these strengths, we also identified gaps that warrant further discussion.

### 4.1. Intervention Gaps and Implications for Future Research

#### 4.1.1. Targeting Multiple Health Behaviors

Studies in this review promoted diet and physical activity using behavior change techniques such as health education, goal-setting, and hands-on teaching, which have been shown to lead to significant improvements in health behaviors in family-based obesity prevention interventions [33]. However, few interventions targeted sedentary behaviors including screen time and sleep behaviors. This finding is consistent with previous reviews that have also reported that sedentary pursuits such as screen use and sleep are not well represented in family-based obesity prevention interventions [19,34]. With the emergence of research and guidelines regarding 24-h activity and sleep behaviors, it is becoming more recognized that there is a need to simultaneously target multiple health behaviors to comprehensively promote healthy lifestyles and reduce obesity [35]. Few studies included in this review (n = 4; 31%) reported statistically or clinically significant reductions in BMI or BMIz score, which may be due to the narrow focus of interventions on diet or physical activity. Our findings are similar to a recently published systematic review examining obesity prevention interventions in Hispanic families that also found that just three studies (33%) reported reductions in BMI or BMIz score [36]. The authors similarly concluded that a broader focus on screen time behaviors and sleep in addition to diet and physical activity is needed to observe a greater intervention effect [36]. 

#### 4.1.2. Lack of Studies with Adolescents

Similar to previous reviews of family-based obesity interventions, our findings also demonstrated that there is a lack of studies focused on the adolescent age range ≥14 years of age [19,36,37]. Adolescence is a critical life period for disease prevention given that youth are undergoing pubertal changes to body composition, hormones, and a precipitous decrease in healthy behaviors like physical activity [38]. Furthermore, the health behaviors established in adolescence have been shown to track into adulthood, impacting long-term disease outcomes [38]. Adolescence is also a life period where youth begin to feel more confident in their own decision-making skills and begin moving toward achieving true behavioral autonomy, further underscoring the importance of focusing on this age group [39]. Many studies have focused on early childhood given the large body of evidence demonstrating the need for early intervention; however, intensive family-based lifestyle interventions among Hispanic adolescents can improve health behaviors and lead to significant reductions in body mass index and risk factors associated with subsequent obesity-related diseases [40]. Given the importance of this life stage for disease prevention, there is a need for more family-based studies to focus on this high-risk age group.

#### 4.1.3. Theoretical Mechanisms

While most studies were theoretically driven, there was great variability in the theories used. Only two studies assessed theoretical constructs, primarily from the SCT and FST, and only one study examined theoretical constructs as mediators, making it difficult to discern which theoretical constructs are most appropriate for this population. For example, the SCT was the most widely used theory in this review and has been reported as the most widely used theory in family-based obesity interventions, including those implemented among Hispanics [41,42,43]. However, previous family-based studies have reported that constructs within the SCT, mainly social support and self-efficacy, do not mediate study outcomes in the context of family-based interventions given that these constructs are individually-focused [44,45]. To assess the appropriateness of theoretical constructs for this population, investigators should more carefully consider the selection, operationalization, and evaluation of behavior change theories among Hispanic youth [44,46]. More adaptable theories that incorporate elements from a broader range of theoretical constructs may be needed to meet the needs and sociocultural context of Hispanic youth and families [47,48,49]. Several studies in this review used multiple theoretical frameworks, yet investigators did not discuss the integration or operationalization of these theories within the intervention. Future studies should provide more detailed information on the behavior change theory or theories used, include an assessment of theoretical constructs, and examine these constructs as mediators when appropriate. This information will provide a greater understanding of the underlying mechanisms by which an intervention is effective, which will guide future family-based obesity prevention efforts in this population [43,50,51].

#### 4.1.4. Defining and Evaluating Family

In addition to lack of guidance on theoretical frameworks, there is also a lack of guidelines on implementing interventions among families [17,18]. As a result, we observed great variability in how ‘family’ was defined and evaluated. For example, in one study, all family members living within the household were invited to attend intervention sessions, while others focused specifically on parent-child dyads, and many did not provide any description of family members included in the intervention. Given the cultural significance of family among this population, how a family operates and is defined has cultural implications that influence health behaviors and outcomes [52]. Furthermore, there is heterogeneity in family processes across Hispanic sub-groups [53,54], and cultural beliefs and traditions in family formation and processes can also change over time. For example, acculturation can lead to changes in family processes within Hispanic families living in the U.S. [55]. Unfortunately, only three studies in this review measured acculturation, limiting our understanding of how acculturation impacted family participation or health behaviors and outcomes. Methods such as qualitative interviews can be used to increase our understanding of family processes around health behaviors in youth and intervention participation in this population [36,56].

In addition to how family is defined, there is also a need to understand who and what should be evaluated at the family-level. Many studies in this review included parent outcomes; however, this was often limited to demographic surveys and few studies assessed self-reported diet in parents or health-related parenting practices (i.e., parenting strategies for eating and activity scale, role modeling health behaviors). Parents who experience weight loss and improvements in dietary habits are more likely to implement changes at the family-level, which can lead to similar changes within the child [57]. Given that an intervention’s effect on the child may be mediated by outcomes and behaviors in participating family members, it is critical for future studies to more clearly describe family engagement and evaluation. Additionally, investigators should consider the evaluation of additional family-level contextual factors that may impact obesity-related outcomes and behaviors [56]. For example, O’Connor et al., assessed family functioning as it has been shown that improving a family’s ability to work and communicate together can improve the family’s ability to participate and make changes to health behaviors as a family unit [58,59]. Similar reviews of family-based obesity prevention strategies have also reported that studies to date do not adequately examine how family factors mediate treatment effects [36,37]. The inclusion and evaluation of multiple family members can increase the complexity of the study design and present statistical challenges. However, this information will increase our understanding of family-level factors that influence obesity and health behaviors in Hispanic youth that should be addressed in future interventions [60].

#### 4.1.5. Acknowledging SDoH

Hispanic youth are disproportionately impacted by SDoH that have been associated with increased rates of obesity and obesity-related diseases [61,62,63]. Thus, intervention strategies should consider the SDoH that may limit disease prevention opportunities and health behaviors that may be leveraged or addressed within interventions to increase engagement and adherence [64]. About half of the articles included in this review addressed SDoH through content focused on determinants such as immigration stress and access to care or by having research staff call families to discuss barriers to program participation, language barriers, and acculturation [58,65,66,67]. For example, Crespo et al. used group sessions with promotoras and clinic providers to improve patient-provider communication and address barriers to care [67]. Addressing SDoH often requires stakeholder engagement and multi-sector collaboration [25,26]. While the majority of studies in this review collaborated with stakeholders for implementation, only about half of the studies integrated SDoH. Partnering with existing community organizations that are trusted entities, embedded within the community is one strategy for leveraging collaboration to address SDoH [25]. For example, we have previously shown that a collaboration between a research institution, local YMCA, and community-based clinic to address access to care in the context of a family-based lifestyle intervention [68] led to establishing care among previously uninsured community members, improve patient-provider relationships, and foster trust within the medical home [68]. Acknowledging and addressing SDoH that negatively impact health behaviors and intervention participation has the potential to increase recruitment, reduce attrition, and increase effectiveness among Hispanic youth and families [36].

Interestingly, all of the studies included in this review that utilized promotoras/community health educators for implementation also used strategies to consider SDoH. Promotoras are lay community health workers who are trained to provide health services to community members. They are members of the Spanish-speaking communities in which they serve, are familiar with Hispanic cultural values and community norms, and have personal insight into barriers to research engagement and behavior change [69]. Qualitative work conducted among Hispanic participants and providers following a family-based childhood obesity intervention revealed that promotoras impact obesity interventions through: (1) personal qualities such as kindness and caring for participants’ families, (2) shared experiences between the promotora(s) and participants, (3) fostering a positive and trusting environment which is critical for populations who lack trust in researchers, (4) encouraging families to engage in research programs and motivating the long-term maintenance of lifestyle changes, and (5) building self-efficacy to advocate for health in clinical settings [70]. Given their community presence and knowledge, leveraging promotoras is a recommended approach for enhancing the utilization of community resources to address SDoH and provides a model for implementation that can be translated in Hispanic communities [71].

#### 4.1.6. Strengths and Limitations

This study focused on a high-risk population that is traditionally underrepresented in research, significantly contributing to the limited body of research describing family-based obesity prevention interventions among Hispanics in the literature [36,72]. A rigorous, comprehensive search strategy across numerous databases was used identify articles. Furthermore, a systematic, in-depth data extraction process was performed in duplicate to ensure reliability. Despite these strengths, this review has several limitations. First, our search was limited to studies written in English, therefore, it is possible that a few otherwise eligible studies from Spanish-speaking countries were missed. For example, our review included only one study from Mexico that provided an English translation. A more in-depth analysis of this study revealed that the investigators included a very high level of engagement among schoolteachers, staff, and board members, compared to studies conducted in the U.S. This suggests that engagement at the school level may be important for effectively reaching Hispanic youth and families. Further exploration of family-based studies in Spanish-speaking countries like Mexico, may lead to other important insights regarding cultural components and implementation strategies that may be applicable to studies conducted in the U.S. focused on Hispanic youth and families. It is also important to note limitations in our review of study results and outcomes given that this review included feasibility studies, pilot studies, and randomized controlled trials, not all studies were adequately powered to detect significant changes in outcomes. Additionally, we did not assess intervention effectiveness or quality as this is more in line with a systematic review and nost a scoping review. A recent systematic review of family-based obesity prevention interventions among Hispanic children has been recently published by Tamayo et al. [36]. It also important to note that data were extracted directly from the articles included in this review and we did not review previously published works or protocol papers. As a result, information for some of the data extraction categories may exist in other works; however, it was not included in this review. It is also important to note that this review included several pilot studies. Given the limited design and sample size that is appropriate for pilot studies, not all of the information for the data extraction categories (i.e., Examined theoretical mediators) may be available within pilot studies. Lastly, the results of this study may be influenced by the search terms that were used, the number databases searched, and the selection of databases used in the search. As a result, this review may be subject to publication bias.

## 5. Conclusions

Given that only 13 studies were identified for this review, there is still a dearth of research focused on using family-based approaches to address obesity among Hispanic youth and families [36]. Most studies included in the review engaged and retained high-risk families in a culturally-adapted, theoretically driven RCT, providing support for this strategy among this population. Given the growing disparities in obesity and obesity-related diseases in this population, it is important to understand the underlying mechanisms by which prevention strategies are effective and engaging among Hispanic youth and families [16]. This requires stronger evaluation and testing of theoretical constructs, family contextual factors, and greater acknowledgement of SDoH that impact health outcomes and behaviors. Community collaborations, including formative work among the population of interest, are needed to gain a deeper understanding of family-level processes that influence health behaviors and priority determinants that should be addressed through prevention strategies. This information will guide the future development and implementation of family-based obesity prevention interventions focused on addressing obesity disparities and promoting health equity.

## Figures and Tables

**Table 1:** Intervention characteristics (n = 13).

	n (%)
Geographic Region	
United States	12 (92.3%)
Mexico	1 (7.6%)
Study Design *	
RCT	9 (69.2%)
Quasi-Experimental	4 (38.5%)
Intervention Setting *	
Home	2 (15.4%)
Primary care/health clinic	3 (23.1%)
Community Organization	2 (15.4%)
School	2 (15.4%)
University	1 (7.7%)
Multi-setting	1 (7.7%)
Not specified	2 (15.4%)
Length of Intervention	
≤10 weeks	3 (23.1%)
11–52 weeks	7 (53.8%)
>53 weeks	3 (23.1%)
Health Behaviors Targeted *	
Diet	12 (92.3%)
Physical Activity	9 (69.2%)
Screen Use	2 (15.4%)
Sleep	1 (7.7%)
Sedentary Behaviors	6 (46.2%)
Theoretical Framework *	
Social Cognitive Theory	6 (46.2%)
Transtheoretical Model of Behavior Change	1 (7.7%)
Family Systems Theory	2 (15.4%)
Behavioral Choice Theory	1 (7.7%)
Food Preference Theory	1 (7.7%)
Socioeconomic Model for Latino Health Promotion	1 (7.7%)
Sociocultural approach	1 (7.7%)
Cross-cultural psychology	1 (7.7%)
Family resilience approach	1 (7.7%)
Health Belief model	1 (7.7%)
Structural model of health behavior	1 (7.7%)
Ecological Model	1 (7.7%)
Not stated	3 (23.1%)
Focus of Evaluation *	
Child	13 (100%)
Parent	11 (84.6%)
Retention Rates	
0–70%	4 (30.8%)
71–80%	4 (30.8%)
81–90%	4 (30.8%)
≥91%	1 (7.7%)

* Indicates that categories are not mutually exclusive, and total may exceed 100%.

**Table 2:** Sample characteristics within interventions (n = 13).

	n (%)
Age of Target Child *	
5–10 years	13 (100%)
11–13 years	9 (69.2%)
Sample Size	
0–100	4 (30.8%)
101–200	2 (15.4%)
201–300	3 (23.1%)
>300	4 (30.8%)
Family Socioeconomic Status	
Low Socioeconomic Status	8 (61.5%)
Not Specified	5 (38.5%)
Family Engagement	
Parent-child dyad	3 (23.1%)
Whole Family	10 (76.9%)
Measured Acculturation	
Yes	3 (23.1%)
Not Measured	10 (76.9%)
Hispanic Subgroups *	
Mexican/Mexican American	10 (76.9%)
Central American	3 (23.1%)
Latin America	2 (15.4%)
Latino/Hispanic Non-specific	5 (38.5%)
Other	4 (30.8%)

* Indicates that categories are not mutually exclusive, and total may exceed 100%.

**Table 3:** SDoH integrated and stakeholder collaboration in interventions (n = 13).

	n (%)
Formative Work with Stakeholders	
Yes	7 (53.8%)
Not Specified	6 (46.2%)
Program Implementers	
Physicians/Providers	4 (30.8%)
Health Educators/Promotoras	6 (46.2%)
Trained Research Staff	4 (30.8%)
Language	
Spanish	3 (23.1%)
Bilingual	8 (61.5%)
Not specified	2 (15.4%)
Cultural Adaptations	
Yes	9 (69.2%)
Not Specified	4 (30.8%)
Acknowledged SDoH	
Yes	6 (46.2%)
Not Specified	7 (53.8%)

## Data Availability

No new data were created or analyzed in this review. Data sharing is not applicable to this article.

## Supplementary Materials

The following are available online at https://www.mdpi.com/article/10.3390/nu13082690/s1, Table S1: Search strategy used to identify eligible family-based childhood obesity prevention interventions among Hispanic youth and families, Table S2: Data extraction categories and availability of data within each article included in the review (n = 13).
