# Supplementary material for: Family-Based Obesity Prevention Interventions among Hispanic Children and Families: A Scoping Review
Source: Nutrients. 2021 Aug 3;13(8):2690. doi: 10.3390/nu13082690 (PMC8402012; doi:10.3390/nu13082690)
Supplement: Supplementary file 1 [file nutrients-13-02690-s001.zip › nutrients-1281169-supplementary.pdf]

Table S1: Search strategy used to identify eligible family-based childhood obesity prevention interventions among Hispanic youth and families.

1.	obesity/ or pediatric obesity/ or overweight/	182,691
2.	(obes* or overweight* or "over-weight*").ti,ab,kw.	291,699
3.	1 or 2	327,553
4.	prevention*.fs.	1,221,210
5.	(prevent* or interven* or program*).ti,ab,kw.	2,674,513
6.	4 or 5	3.439.168
7.	pediatrics/	50,955
8.	adolescent/ or child/	2,672,157
9.	(child* or pediatric* or paediatric* or kid* or adolescen* or teen* or preteen* or "pre-teen*" or tween* or juvenil* or youth*).ti,ab,kw.	2,132,059
10.	7 or 8 or 9	3,756,377
11.	exp Hispanic Americans/	29,702
12.	(hispanic* or mexican* or latin* or spanish* or cuba* or "puerto ric*" or "south americ*").ti,ab,kw.	179,092
13.	11 or 12	184,013
14.	parents/ or mothers/ or fathers/ or family	169,077
15.	((parent* or mother* or father* or mom* or dad* or family* or families* or familia* or communit* or home*) adj3 (focus* or orient* or involv* or partic* or complet* or base*)).ti,ab,kw.	186,387
16.	14 or 15	332,797
17.	3 and 6 and 10 and 13 and 16	605

Table S2. Data extraction categories and availability of data within each article included in the review (n=13).

Author (year)	Study Design	Intervention Participants	Study Duration	Setting	Socioeconomic Status	Sample Size	Geographic Location	Retention	Retention Strategies	Theoretical Framework	Health Behaviors Addressed
Falbe (2015)	√	√	√	√	√	√	√	√		√	√
O'Connor (2020)	√	√	√	√	√	√	√	√	√	√	√
Parsons (2019)	√	√	√	√		√	√	√		√	√
Arredondo (2018)	√	√	√	√	√	√	√	√		√	√
Hull (2018)	√	√	√	√	√	√	√	√		√	√
Crespo (2018)	√	√	√	√	√	√	√	√		√	√
Horton (2013)	√	√	√	√	√	√	√	√			√
Hammons (2013)	√	√	√	√		√	√	√		√	√
Wright (2012)	√	√	√	√	√	√	√	√			√
Crespo (2012)	√	√	√	√		√	√	√		√	√
Bacardi-Gascon (2012)	√	√	√	√		√	√	√		√	√
Cronk (2011)	√	√	√	√	√	√	√	√			√
Olvera (2010)	√	√	√	√	√	√	√	√		√	√

Author (year)	Measures	Outcomes	Participant Evaluated	Language	Program Format	Program Implementer	Cultural Adaptations	SDoH Acknowledged	Formative Work	Collaboration for Implementation
Falbe (2015)	√	√	√	√	√	√	√	√	√	√
O'Connor (2020)	√	√	√	√	√	√	√	√	√	√
Parsons (2019)	√	√	√	√	√	√	√			√
Arredondo (2018)	√	√	√	√	√	√	√	√		
Hull (2018)	√	√	√	√	√	√	√		√	√
Crespo (2018)	√	√	√	√	√	√	√	√		√
Horton (2013)	√	√	√	√	√	√	√	√	√	√
Hammons (2013)	√	√	√	√	√	√	√		√	
Wright (2012)	√	√	√	√	√	√	√		√	√
Crespo (2012)	√	√	√	√	√	√	√			√
Bacardi-Gascon (2012)	√	√	√		√	√				√
Cronk (2011)	√	√	√	√	√	√	√	√		√
Olvera (2010)	√	√	√		√				√	√
